# Adipose tissue homeostasis is deeply disrupted by doxorubicin treatment

**DOI:** 10.1186/2049-3002-2-S1-P5

**Published:** 2014-05-28

**Authors:** Helena Batatinha, Camila Souza, Edson Lima, Maria Isabel Alonso-Vale, Maysa Cruz, Roberta Da Cunha, Fabio Lira, Jose Rosa

**Affiliations:** 1 Department of Cell and Developmental Biology, University of São Paulo, São Paulo, Brazil; 2 Department of Biological Sciences, Institute of Environmental Sciences, Chemical and Pharmaceutical, Federal University of São Paulo, São Paulo, Brazil

## Introduction

Doxorubicin is an anthracyclin antibiotic commonly used in chemotherapy against several types of cancer, however it has a great number of side effects [1,2]. Doxorubicin treatment reduces body weight, affects total cholesterol, free fatty acids and the level of fasting blood glucose [3]. Furthermore this chemotherapy can act to damage the adipocytes increasing the development of insulin resistance in peripheral tissues [1]. Our aim was to investigate the doxorubicin effects on adipocyte tissue and its functions.

## Materials and methods

We performed *in vivo* studies with 20 male rats, 10 treated 72 hours before the sacrifice with 15 mg/kg doxorubicin and 10 treated with PBS. We took the retroperitoneal adipocyte tissue to measure lipogenesis, lipolysis, and glucose uptake. We also had an *in vitro* testing using 3T3-L1 cells treated with doxorubicin to perform toxicity and lipolysis assays.

## Results

Our results showed that in rats, lipogenesis was significantly decreased and lipolysis was also decreased. The glucose uptake test was significantly decreased and the free fatty acids were increased. *In vitro* assay showed that higher doses (10 and 100 nM concentrations) were toxic, inducing over 90% cellular apoptosis. Lipolysis *in vitro* was decreased when the cells were treated with 10 nM for 30 minutes; in this interval this concentration was not toxic because the LDH activity was not modified.

## Conclusions

We can conclude that doxorubicin has many effects upon adipocyte tissue homeostasis, decreasing lipolysis and lipogenesis, glucose uptake and inducing cellular apoptosis in high concentrations, being able to change its function. We are still investigating the molecular mechanisms of this process.
